# The use of scratch-cards to increase alcohol consumption awareness and facilitate a reduction

**DOI:** 10.1186/1940-0640-8-S1-A12

**Published:** 2013-09-04

**Authors:** John Bradley, Craig Jones, Sarah Jones, Carol Foster, Paul Jordan

**Affiliations:** 1 Public Health Wales, Cardiff, UK

## 

Alcohol misuse is linked to over 60 diseases and harmful conditions, from acute related harm such as serious injury to risky sexual practice and long term chronic mental and physical health issues such as liver disease and the early onset of dementia. In Wales, harm caused by alcohol costs the NHS over £85 million per year and is responsible for 1 in 30 deaths. A national programme of brief intervention training is taking place across Wales to impact the culture of hazardous and harmful drinking across Wales. In order to ensure a population approach, Public Health Wales is developing a basic scratch-card so drinkers can easily keep track on how much alcohol they consume and receive some advice and information on their of consumption. Scratch-card will be made available for free and drinkers will 'log/scratch' their intake. This will be monitored over a period of months by various health and social care professionals delivering Alcohol Brief Interventions to see if there is any reduction in their client's drinking patterns. The pilot project will be rolled out over the winter 2013. It is hoped that the scratch-card will help reduce hazardous and harmful drinking and reduce the impact on services due to alcohol.

